# Paraneoplastic neuromyelitis optica associated with ANNA-1 antibodies in invasive thymoma

**DOI:** 10.1186/1471-2415-14-106

**Published:** 2014-09-03

**Authors:** Hee Kyung Yang, Se Joon Woo, Woong-Yang Park, Jeong-Min Hwang

**Affiliations:** 1 Department of Ophthalmology, Seoul National University College of Medicine, Seoul National University Bundang Hospital, 166 Gumiro, Bundang-gu, 463-707 Seongnam, Gyeonggi-do, Korea; 2 Samsung Genome Institute, Samsung Medical Center, Seoul, Korea; 3 Department of Molecular Cell Biology, Sungkyunkwan University School of Medicine, Suwon, Korea

**Keywords:** Paraneoplastic neuromyelitis optica, Thymoma, Antineuronal nuclear antibody

## Abstract

**Background:**

Thymoma is associated with various paraneoplastic autoimmune disorders. Herein, we report paraneoplastic neuromyelitis optica (NMO) associated with both anti-aquaporin-4 (AQP4) immunoglobulin G (IgG) and type 1 antineuronal nuclear antibody (ANNA-1) in an invasive thymoma patient.

**Case presentation:**

A woman presented with a sudden onset of bilateral progressive visual loss associated with recurrence of invasive thymoma, 6 years after thymectomy and immunosuppressive treatment. AQP4-IgG and ANNA-1 were present in the patient’s serum. Visual outcome was poor despite immunosuppressive treatment. Longitudinally extensive transverse myelitis developed 27 months after the onset of visual symptoms.

**Conclusions:**

We have reported a case of paraneoplastic NMO associated with both AQP4-IgG and ANNA-1 autoantibodies in an invasive thymoma patient.

## Background

Thymoma is associated with various paraneoplastic autoimmune disorders [1]. However, there has been no report of paraneoplastic neuromyelitis optica (NMO) associated with both anti-aquaporin-4 (APQ4) immunoglobulin G (IgG) and type 1 antineuronal nuclear antibody (ANNA-1) in an invasive thymoma patient. We found a patient with invasive thymoma who developed bilateral progressive visual loss and showed circulating APQ4-IgG and ANNA-1 autoantibodies. Lower extremity weakness and tingling sense developed 27 months after the onset of visual symptoms, and she was finally diagnosed as definite NMO.

## Case presentation

**Figure 1:**
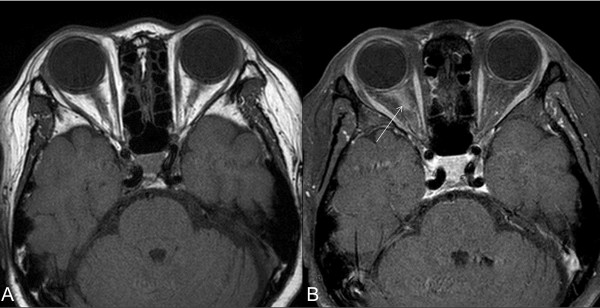
**Brain magnetic resonance imaging 1 week after clinical onset of visual loss in the right eye.** Axial fat saturated T1-weighted imaging (T1WI) **(A)** and contrast-enhanced T1WI **(B)** shows enhancement of the intraorbital portion of the right optic nerve (white arrow).

A 55-year-old woman showed a sudden onset of painless progressive vision loss in her right eye for 1 week. Six years ago, she underwent thymectomy for invasive thymoma (WHO classification B2) followed by chemotherapy (adriamycin, cisplatin, vincristine, and cyclophosphamide) and radiation therapy. The thymoma had completely regressed following chemoradiation, and no metastasis or recurrence were detected during follow-up examinations.On ocular examination, her visual acuities were hand motion in the right eye and 20/20 in the left eye, with a relative afferent pupillary defect in the right eye. The anterior segment and media of both eyes were normal. Intraocular pressure of both eyes were 14 mmHg. Ocular motility was normal without any pain during extraocular muscle movements. Ptosis or exophthalmos were absent in either eye. Fundus examinations of the optic disc and retina were unremarkable. She could not recognize the demonstration plate of Ishihara color vision test and any of the Hardy, Rand, and Rittler (HRR) color vision test with the right eye, but could recognize all of the Ishihara color vision test and HRR color vision test with the left eye. Goldmann perimetry showed a small nasal island in the right eye. Magnetic resonance imaging (MRI) revealed an abnormal contrast-enhancement of the right optic nerve extending to the prechiasmatic portion, suggesting an acute inflammatory or demyelinating process (Figure 1). No treatment was administered owing to the patient’s refusal. After 2 months, visual acuity and visual field defects of the right eye showed a further gradual deterioration without recovery and the patient was lost to follow-up.

The patient returned to our hospital after 5 months, presenting with a sudden onset of visual acuity decrease in the left eye for 1 week. In the right eye, she had visual acuity of light perception, and the visual acuity of the left eye had reduced to 20/30 from 20/20. Color vision tests revealed a moderate red-green and blue-yellow color defect in the left eye. Visual field testing revealed a generalized reduction of sensitivity and a cecocentral scotoma in the left eye. The anterior segment and media of both eyes were normal. Funduscopy showed total pallor of the optic disc in the right eye, but a normal optic disc without edema or pallor in the left eye. Visual evoked potentials were delayed in both eyes. MRI of the orbit and brain revealed high signal intensities of both optic nerves on T2-weighted images and increased abnormal enhancement of the right optic nerve extending to the prechiasmatic portion. However, there was no evidence of brain metastasis or cerebrospinal fluid (CSF) seeding.

Systemic evaluation was performed to investigate the presence of malignancy; chest computed tomography (CT) revealed a 4.7-cm nodule in the left lower lobe and lung biopsy confirmed the diagnosis of a malignant epithelial neoplasm with cytokeratin expression, no epithelial membrane antigen, no CD5 expression, and no neuroendocrine marker expression. It was histopathologically similar to her prior thymoma and the possibility of recurrent thymoma was considered. Whole body positron emission tomography showed no other areas of abnormal hypermetabolic lesions. Serological examination for major paraneoplastic autoantibodies revealed the presence of APQ4-IgG and ANNA-1, while anti-Yo antibody, ANNA-2, anti-Ri, anti-acetylcholine receptor antibodies, anti-recoverin, anti-alpha-enolase, and anti-collapsin response-mediator protein-5 (CRMP5) antibodies were absent. Blood analysis, including erythrocyte sedimentation rate, C-reactive protein, thyroid function tests, and angiotensin-converting enzyme levels were within normal range, and tumor markers (CEA, CA19-9, and CA125) were absent. Microbiological tests were negative for varicella zoster virus, cytomegalovirus, Epstein-Barr virus, syphilis, and HIV. Antinuclear antibody, anti-double stranded DNA and anticardiolipin antibodies were negative. Cytological examination of the CSF showed no abnormal findings, including oligoclonal bands. Primary mutations for Leber’s hereditary optic neuropathy of 11778/ND4, 3460/ND1, 14484/ND6 and 4171/ND1 were absent.

On the basis of the clinical presentation, neuroimaging, and presence of autoantibodies, her condition was diagnosed as NMO spectrum disorder associated with invasive thymoma. Intravenous methylprednisolone was administered, followed by oral prednisolone (60 mg) and oral cyclosporine (600 mg) daily for 2 weeks. However, her visual acuities continuously deteriorated to no light perception in the right eye and counting fingers in the left eye. After 6 weeks of onset in her left eye, optical coherence tomography showed diffuse atrophy of the retinal nerve fiber layers in both eyes, but the outer retina and photoreceptor layers were relatively intact. Standard electroretinograms (ERG) were normal in both eyes, but multifocal ERG (mfERG) showed a decreased response in the macula of both eyes. Follow-up MRI examination revealed increased abnormal enhancement of the left optic nerve. The patient received 6 cycles of chemotherapy with etoposide, paclitaxel, and prednisolone over the next 6 months and was maintained with azathioprine. The left lower lung mass had regressed on follow-up chest CT; however, visual acuities were unchanged. No evidence of tumor recurrence or neurologic signs of limb weakness and sensory abnormalities occurred up to 13 months after chemotherapy, when suddenly, she developed symptoms of lower limb weakness and tingling sense. Neurologic examination revealed proximal dominant weakness in both lower extremities of grade 4. Tingling sense below sensory level T10 developed with pain/temperature, vibration and positional sense hypesthesia. Whole spine MRI revealed longitudinally extensive transverse myelitis with central T2 high signal intensity of the spinal cord from level T3/4 to T11. She was diagnosed as definite NMO and her myelitis improved after administration of intravenous methylprednisolone.

## Discussion

Thymoma has been associated with several immunologically mediated diseases, and various paraneoplastic autoantibodies [1]. Although rare, thymoma associated with visual symptoms has been reported in association with paraneoplastic autoantibodies, such as cancer-associated retinopathy (CAR) with anti-retinal antibodies, paraneoplastic optic neuropathy (PON) with anti-CRMP5 antibody, or NMO with AQP4-IgG [2]. However, there has been no report of the combined presence of APQ4 and ANNA-1 autoantibodies. Our patient showed predominant involvement of the retinal ganglion cells associated with severe retinal nerve fiber layer thinning, while the outer retina and photoreceptor layers were relatively intact. Standard ERG were normal in both eyes. MfERG responses were slightly depressed in the center. However, for patients with poor fixation, the accuracy of mfERG results may be difficult to interpret [3]. Particularly, central mfERG amplitudes are most affected by unsteady fixation as in our patient. Therefore, there was no evidence to suspect retinal abnormalities [3]. The abnormal contrast-enhancement of the optic nerve on MRI suggested an inflammatory or demyelinating pathology.

APQ4-IgG is known to be highly specific for NMO with specificity reported up to 94% [4]. An earlier study conducted before the discovery of APQ4-IgG has reported central nervous system and thymic epithelial cell–specific antibodies in a patient with thymoma and myasthenia gravis during the acute phase of NMO, suggesting that the NMO was linked to the thymoma [5].

Transverse myelitis developed 2 years after the onset of visual loss, which is not typical for definite NMO historically with a monophasic course of bilateral optic neuritis and myelitis with short intervals between the index events [4]. The long interval between events may be attributed to the long-term immunosuppression and chemotherapy that may have decreased APQ4-IgG titers, thus preventing further relapse or development of myelitis during the course of the disease. Weinshenker et al [6] have reported the importance of serum AQP4-IgG in the disease progression of NMO patients, and suggested that immunosuppressive treatment may lessen the chance of a second or subsequent attack of transverse myelitis even before the clinical criteria for NMO are satisfied. On the other hand, Matiello et al [7] have shown that after a median of 9 years of follow-up, 50% of APQ4-IgG seropositive patients with initial optic neuritis may experience an episode of myelitis and fulfill the criteria of denite NMO. Therefore, a longer follow-up for several years may reveal a subsequent myelopathy as in our case. A long-term follow-up of serum autoantibodies as well as serial neurologic examinations is warranted in such cases.

The clinical significance of APQ4-IgG associated with cancer is unclear. APQ4-IgG was found to be associated with cancer in 0.004% of cases, including 1 thymoma patient screened for paraneoplastic autoantibodies [8]. Six of these patients had NMO spectrum disorders before or after cancer detection (3–18 months), whereas 2 did not have any neurological evidence of an NMO spectrum disorder [8]. A previous study has also reported serum positive APQ4-IgG in a patient with possible paraneoplastic NMO associated with lung cancer [9]. Therefore, the clinical utility of this autoantibody as a cancer marker warrants prospective investigation.

ANNA-1 is recognized as an IgG marker for small-cell lung carcinoma, and has rarely been found in thymoma patients with neurologic autoimmunity. The oncologic implication of ANNA-1 seropositivity has been shown in a previous report of 172 thymoma patients, where 3% of cases were positive for ANNA-1 [10]. However, isolated visual symptoms have not been reported with ANNA-1, and thus it may not be directly related to optic neuropathy. The presence of paraneoplastic NMO-associated serum APQ4-IgG alone may lead to a poor visual prognosis which is refractory to immunosuppressive treatment. Thus, the implication of the coexistence of ANNA-1 remains to be elucidated.

## Conclusion

Thymoma could be associated with paraneoplastic NMO with both APQ4-IgG and ANNA-1 autoantibodies.

## Consent

Written informed consent was obtained from the patient for publication of this case report and any accompanying images. A copy of the written consent is available for review by the Editor of this journal.

## Competing interests

The authors declare that they have no competing interests.

## Authors’ contributions

The work was carried out in collaboration between all authors. All authors read and approved the final manuscript.

## Pre-publication history

The pre-publication history for this paper can be accessed here:

http://www.biomedcentral.com/1471-2415/14/106/prepub
